# Heart Failure and Atrial Fibrillation: From Basic Science to Clinical Practice

**DOI:** 10.3390/ijms16023133

**Published:** 2015-01-30

**Authors:** João Pedro Ferreira, Mário Santos

**Affiliations:** 1 Internal Medicine Department, Centro Hospitalar do Porto, Porto 4099-001, Portugal; 2 Department of Physiology and Cardiothoracic Surgery, Cardiovascular Research and Development Unit, Faculty of Medicine, University of Porto, Porto 4200-319, Portugal; E-Mail: mariossantos001@gmail.com; 3 Cardiology Department, Centro Hospitalar do Porto, Porto 4099-001, Portugal

**Keywords:** atrial fibrillation (AF), heart failure (HF), pathophysiology

## Abstract

Heart failure (HF) and atrial fibrillation (AF) are two growing epidemics associated with significant morbidity and mortality. They often coexist due to common risk factors and shared pathophysiological mechanisms. Patients presenting with both HF and AF have a worse prognosis and present a particular therapeutic challenge to clinicians. This review aims to appraise the common pathophysiological background, as well as the prognostic and therapeutic implications of coexistent HF and AF.

## 1. Introduction

In developed countries, heart failure (HF) affects 2% to 3% of the population and is a major cause of morbidity and mortality [1]. Despite the therapeutic progress observed in past decades, the prognosis of HF patients remains poor [2]. Atrial fibrillation (AF) is the most common heart rhythm disorder with an overall prevalence of 1% [3]. Similarly to HF, it is also associated with significant morbidity, mortality and an economic burden [4]. These two diseases often coexist because they share common risk factors (older age, hypertension, diabetes mellitus, valvular and ischemic heart disease) and pathophysiological mechanisms. In addition, they can promote each other by inducing neuro-hormonal, electrophysiological and hemodynamic changes. Notably, the development of the second is associated with a worse prognosis regardless of which condition comes first [5]. There are several specific therapeutic implications to each disease when they coexist.

This review aims to appraise the common pathophysiological background, as well as the prognostic and therapeutic implications of coexistent HF and AF.

## 2. Combined Heart Failure and Atrial Fibrillation: Epidemiological and Prognostic Implications

Among HF trials and registries, the prevalence of AF ranged from 13% to 41%, depending in part upon age and the severity of HF [5,6], with no differences between heart failure with preserved or reduced ejection [7]. Conversely, the prevalence of HF in recent trials involving AF patients varied from 30% to 65% [8,9]. In reference to their temporal relationship, Framingham cohort study [5] showed that the frequency of HF preceding AF was similar to AF preceding HF.

The prognostic importance of the presence of AF in HF patients is well established in different settings. Both observational studies [5] and randomized clinical trials [6,10] showed that the presence of AF was associated with increased hospitalization, hospital stay and mortality of HF patients. A recent meta-analysis that included more than 30,000 HF patients showed that those with AF had a 33% increase in mortality [11].

Together, these data show that HF and AF often coexist and when together they are associated with worse prognosis.

## 3. Common Pathophysiological Background for Heart Failure and Atrial Fibrillation

### 3.1. Hemodynamic Mechanisms

An increased left ventricular filling pressure (LVFP) is a hallmark feature of the HF hemodynamic profile, which can be caused by either a systolic or diastolic dysfunction [1]. This increased LVFP is transmitted to the left atrium, which will lead to several macro- and microscopic changes in this chamber. The elevated atrial pressure is further increased when functional mitral regurgitation develops along the LV remodeling. This increased stress in the atrium wall is mechanotransduced and will drive several of the cellular and molecular mechanisms discussed below.

**Figure 1:**
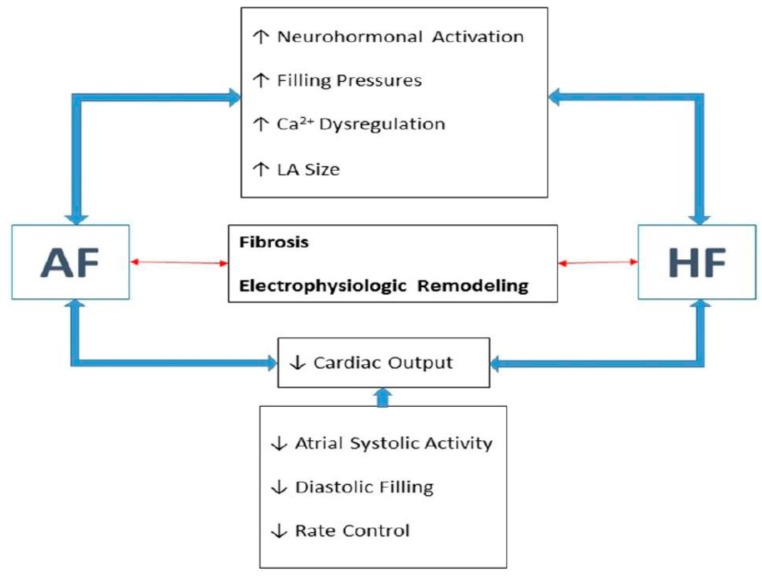
Common pathophysiological background for heart failure (HF) and atrial fibrillation (AF). LA: left atrial.

On the other side, AF can interfere with the ability of the heart to pump or accommodate blood. An increased resting heart rate and an exaggerated hear. rate response to exercise shorten the LV filling time. Together with the concomitant loss of an effective atrial contraction, AF can significantly compromise diastolic function. In addition, a sustained rapid heart rate can impair systolic function by reducing myocardial contractility [12] (Table 1 and Figure 1).

**Table 1:** Common pathophysiological mechanisms of heart failure and atrial fibrillation.

Items	Pathophysiological Mechanisms
Hemodynamic	Increased left ventricle filling pressure
	Increased resting heart rate
	Exaggerated heart rate response to exercise
	Loss of atrial contraction
	Reduced myocardial contractility
Neuro-hormonal	Renin-angiotensin-aldosterone system activation
	Adrenergic activation
	Increase of transforming growth factor-β1
Cellular	Extracellular matrix alteration
	Intracellular calcium overload

### 3.2. Neuro-Hormonal Mechanisms

Atrial stretch results in an increased neurohormonal activation. The renin-angiotensin-aldosterone system (RAAS) activation enhances signal transduction of downstream pathways such as mitogen-activated protein kinase (MAPK) [13,14,15], Janus kinase (JAK)/signal transducers and activators of transcription (STAT) [15], transforming growth factor-β1 (TGF-β1) [16,17], and angiotensin II activated platelet-derived growth factor-A (PDGF-A) pathways [18], which play an important role in fibrosis formation and cardiac remodeling. Additionally, increased levels of Rac1—a small guanosine triphosphate-binding protein, and nuclear factor-kappa B (NF-κB)—a transcription factor, are increased in AF tissues [19,20]. Rac1 may itself activate NF-κB [21] and STAT [22], and angiotensin II can activate all these signaling pathways [23]. Activation of angiotensin II type 1 (AT-1) receptors initiates a cascade of phosphorylation processes that activate a family of mitogen-activated protein kinases (MAP kinases) that promote atrial hypertrophy, fibrosis, and apoptosis, contributing to the structural remodeling of this heart chamber [24]. The stimulation of AT-1 receptors also activates phospolipase C leading to inositol-1,4,5-triphosphate (IP3) that mediates the release of calcium from the sarcoplasmic reticulum which can have pro-fibrotic and arrhythmogenic effects [25]. Enhanced left ventricular wall stress also increases neurohormonal activation resulting in myocardial hypertrophy [26] and interstitial remodeling [27]. Transforming growth factor β1 is involved in maladaptive remodeling [28] and insulin-like growth factor 1 results in adaptive remodeling [29]. Matrix metalloproteinases that degrade extracellular matrix proteins can increase ventricular remodeling in HF. Adrenergic activation, an important feature of HF [30] may also be impact on AF pathophysiology. There are multiple lines of evidence linking high levels of β1-adrenergic signaling, as predicted for β1 389-arginine homozygotes, to the development of AF [31]. Higher adrenergic activity has been shown to increase the inducibility of AF in a dose-dependent manner [32,33]. Furthermore, in isolated human right atrial preparations, isoproterenol infusion has been shown to increase the frequency of atrial early and delayed after-depolarizations, phenomena that have been implicated in initiating AF [34] (Table 1 and Figure 1).

### 3.3. Cellular and Intra-Cellular Mechanisms

In the interstitial compartment, fibroblasts modify the extracellular matrix with effects on ventricular size, structure, and stiffness. If AF persists, further structural changes occur, promoting volume increase of atrial myocytes, sarcomeres misalignment, accumulation of glycogen, and gap-junctional remodeling [35]. In the presence of HF, the auricular stretch induced by volume overload largely contributes to AF pathophysiology [36]. Furthermore, HF can cause atrial dilatation that serve as a mold able to support a large number of re-entry wavelets that are essential for AF maintenance [7]. In synthesis, HF creates a favorable structural background for atrial re-entry and ectopic activity [7], promoting further arrhythmogenesis [37].

Calcium overload of atrial myocytes occurs early in the development of AF and causes changes in gene expression that down-regulate the l-type calcium current, leading to atrial refractory period shortening in order to compensate for the calcium overload and consequently promoting multiple re-entry [38]. After depolarization, sarcoplasmic calcium is recaptured to the sarcoplasmic reticulum via the calcium ATPase (SERCA2a). In HF, SERCA2a is reduced leading to high cytosolic and low sarcoplasmic reticulum calcium concentrations [39]. Atrial fibrillation itself activates stretch-mediated channels that enhance calcium binding to cellular myofilaments that, in turn, can produce delayed after-depolarisations and triggered activity. Persistent and paroxysmal AF are associated with profound impairment in calcium metabolism [40,41,42]. Increased diastolic sarcoplasmic reticulum calcium leak and related delayed after-depolarizations/triggered activity promote cellular arrhythmogenesis in paroxysmal AF patients. Previous studies suggested that increased calcium uptake resulting from phospholamban hyper-phosphorylation, and ryanodine receptor channel dysregulation by sarcoplasmatic reticulum increased spontaneous cellular activity in paroxysmal AF [43]. These findings provide important evidence for the role of calcium-dependent ectopic activity in paroxysmal AF, which are different from those of long-standing persistent AF patients that have profound alterations in l-type calcium currents and action potential durations [43]. These results provide opportunities to develop tailored therapeutic approaches for AF (Table 1 and Figure 1).

## 4. Fibroblast Growth Factor-23: A Key Link between Chronic Kidney Disease, Atrial Fibrillation and Heart Failure

**Figure 2:**
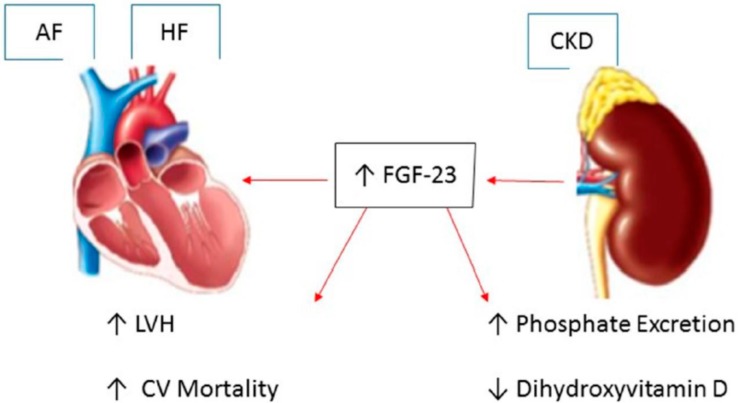
Fibroblast growth factor-23 (FGF-23): A key link between chronic kidney disease, atrial fibrillation and heart failure. CKD: chronic kidney disease; LVH: Left ventricular hypertrophy; CV: cardiovascular; ↑ up-regulation; ↓ down-regulation.

Fibroblast growth factor-23 (FGF-23) is a bone-derived hormone that plays a central role in phosphate homeostasis. FGF-23 acts on the kidney to promote urinary phosphate excretion and to inhibit the production of 1,25-dihydroxyvitamin D, thereby reducing gastrointestinal absorption of dietary phosphate [44]. Circulating FGF-23 concentrations rise substantially with chronic kidney disease (CKD). In human studies, higher circulating concentrations of FGF-23 have been associated with increased left ventricular mass as well as incident heart failure, myocardial infarction, and cardiovascular death [45]. Increased cardiac hypertrophy induced by FGF-23 can lead to diastolic dysfunction and a rise in left ventricular filling pressures, resulting in left atrial dilation and fibrosis, an important structural substract for AF initiation [46]. Data from the Multi-Ethnic Study of Atherosclerosis (MESA) and Cardiovascular Health Study (CHS) showed an association between circulating FGF-23 concentration and incident AF [44]. In multivariable analysis models, each two-fold-higher FGF-23 concentration was associated with a more than 30% AF risk increase. Therefore, higher circulating FGF-23 concentration is associated with incident AF and may partially explain the link between CKD, HF and AF [44] (Figure 2).

## 5. Atrial Structure and Function Influence on Thromboembolic Risk and Heart Failure

Understanding the association between atrial structure and function with thromboembolic and HF risk is very important to improve preventive and therapeutic strategies. The Effective aNticoagulation with factor xA next GEneration in AF-Thrombolysis In Myocardial Infarction 48 (ENGAGE AF-TIMI 48) study [47] evaluated left atrial (LA) size and function, according to the electrical burden of AF (paroxysmal, persistent, and permanent) as well as stroke risk expressed in the CHADS2 score (congestive heart failure, hypertension, age ≥ 75 years, diabetes mellitus, stroke). This study identified strong correlations between increasing abnormalities of LA structure and function with greater burdens of AF and higher CHADS2 score—an estimate of stroke risk. While the majority of AF subjects had LA enlargement, impairment of LA function was also demonstrated among a large number of subjects with normal LA size. These findings suggest that the assessment of LA function may add important information in the evaluation of the AF patient [48], in order to improve stroke risk stratification beyond that achieved with conventional clinical characteristics [49,50,51].

## 6. Obesity and Epicardial Fat Increase Atrial Arrhythmogenesis

Obesity increases the risk of developing HF, ischemic heart disease, and AF [52,53]. Chamber dilatation and hypertrophy are associated with obesity and may explain the increased risk of AF [54]. This epicardial adipose tissue is also associated with AF, presumably due to higher levels of inflammatory mediators, such as adipocytocines [55] and neurally-mediated mechanisms such as vagal modulation [56,57]. The direct contact of epicardial fat with the atria may induce direct atrial arrhythmogenic effects [55,58]. In the context of HF, epicardial fat prolongs LA action potentials duration, increasing calcium influx and LA contractility and triggered activity [59]. Since the epicardial fat is not evenly distributed over the atrial wall, it is possible that the action potentials prolongation effects of epicardial fat may contribute to larger atrial electrical dispersions and facilitate the maintenance of re-entrant circuits [60]. Abnormal epicardial fat has been associated with endothelial dysfunction [61], which in turn is associated with higher risk of stroke [62] and lower probability of conversion to sinus rhythm [63]. Epicardial fat in contact with the LA correlated with levels of soluble intercellular adhesion molecule 1 (sICAM-1) and von Willebrand factor (vWF), suggesting that epicardial adipose tissue may modulate endothelial function in patients with AF possibly through a paracrine mechanism [64].

In contrast to AF, patients with HF were found to have less epicardial fat mass and smaller adipocytes than controls [65], possibly due to systemic and local catabolic derangements and impaired tissue oxygenation in HF [65]. Consequently, the smaller cells size of HF adipocytes would produce lower concentrations of inflammatory cytokines and adipokines [66,67], providing a potential explanation for the better prognosis found in obese HF with reduced ejection fraction patients (HF-REF)—the so-called “obesity paradox”[52,68]. The “obesity paradox” is only observed in obese HF-REF patients. On the other hand, obesity, particularly central and/or visceral adiposity, is independently associated with diastolic dysfunction [69,70,71,72].

## 7. Abnormal Gene Expression in Atrial Fibrillation

The mechanisms underlying susceptibility to most forms of AF remain unknown [73]. Some forms of atrial fibrillation, especially “lone AF” may have a heritable pattern [74,75]. At the molecular level, the onset of HF is associated with reprogramming of gene expression, including down regulation of the α-myosin heavy chain (α-MHC) gene and sarcoplasmic reticulum calcium ATPase genes and reactivation of specific fetal cardiac genes such as atrial natriuretic factor (ANF) and brain natriuretic peptide (BNP) [76]. Additionally, arrhythmias in general are frequent in patients with hereditary myopathies such as laminopathies, Emery-Dreifuss muscular dystrophy, myotonic dystrophy I, mitochondrial myopathies, fatty-acid oxidation defects, and dystrophinopathies which indicate that hereditary myopathies carry an increased risk for developing potentially severe arrhythmias and sudden death. Therefore, close follow-up and long-term rhythm surveillance may prevent fatal complications in these patients [77].

## 8. Heart Failure and Atrial Fibrillation: Treatment Implications

In general, the evidence on HF or AF treatments is generalizable to patients presenting with both diseases because it is unlikely that the proven benefit to one disease disappears when the other is simultaneously present. In addition, most of the trials testing specific treatments to AF or HF included a subset of patients who had both diseases, which further strengthens their external validity to this specific group of patients. Nevertheless, there are some specific therapeutic implications when managing patients with coexistent HF and AF that clinicians should be aware.

As previously discussed, AF is a robust and independent prognostic marker in HF populations. However, the conjectural benefit of rhythm control has never been empirically proved. The Atrial Fibrillation Follow-up Investigation of Rhythm Management (AFFIRM) [78] and the Atrial Fibrillation in Congestive Heart Failure (AF-CHF) [79] trials demonstrated similar all-cause HF incidence, hospitalization and overall mortality in both rhythm control and rate control groups. This discrepancy between the worse outcomes in AF patients compared to those in sinus rhythm is partially indicted to the limited efficacy, as well as to the significant adverse events of the available antiarrhythmic drugs. Other important determinant to this rhythm *versus* rate control decision is the presence of symptoms attributed to AF despite controlled heart rate. Despite some dissent results regarding quality of life (QoL) impact of these treatment strategies [80,81], the lower QoL in AF patients and its recognized detrimental hemodynamic impact legitimate the option for rhythm control in selected symptomatic AF patients. Conversely, it is appropriate to pursue rate rather than rhythm control if symptoms related to AF are deemed acceptable [82].

Several clinical trials have consistently shown the benefits of anticoagulation in AF, which is a powerful risk factor for stroke and thromboembolism. The decision to initiate anticoagulation therapy is adequately informed by thromboembolic risk stratification scores as CHADS2 (congestive HF, hypertension, age, diabetes, stroke) and CHA2DS2-Vasc (congestive HF, hypertension, age, diabetes, stroke, female gender, vascular disease) [82]. These scores assigns one point to each variable, other than age above 75 years or a previous history including a thromboembolic event, which gets two points. Hence, according to these scores HF and hypertension and coronary artery disease (CAD) carry the same thromboembolic risk. However, HF seems to be associated with increased risk than diabetes or CAD [83], especially when LVEF is reduced [52]. Therefore, these scores may underestimate the thromboembolic risk in patients with AF and HF. In practical terms, when the score gives an intermediate risk (1 point), the AF patient who presents isolated HF should be considered at increased risk compared to others having 1-point due to diabetes, CAD or hypertension.

The efficacy of conventional HF drugs in primary prevention of AF remind us how interconnected these diseases are. Angiotensin-converting enzyme inhibitors [84], angiotensin receptor blockers [85], β-blockers [86] and mineralocorticoid receptor antagonists [87] had all been shown to reduce AF incidence in HF patients.

Cardiac resynchronization therapy (CRT) consists of a biventricular pacing in order to restore synchronicity of left and right ventricles activation. Several trials demonstrated a mortality benefit in HF populations, however the presence of AF has been significantly associated with a non-response to CRT [88]. This may be explained by a true smaller effect of CRT in AF patients, which usually are older, have more advanced HF and more comorbidities. An alternative explanation is the suboptimal delivery of biventricular pacing that AF patients are more likely to have because of the loss of biventricular capture due to pseudo-fusion or fusion beats. The underrepresentation of AF in CRT trials and underpowered studies to detect differences in HF populations with AF makes less clear the clinical benefits of CRT in this specific subgroup of patients [89]. Despite the weak evidence, the general opinion is that symptomatic AF patients (class III and IV of New York Heart Association) may benefit from CRT provided that biventricular pacing is close to 100%, using either drugs or atrioventricular junction ablation [90].

## 9. Conclusions

AF and HF are two growing epidemics that often coexist due to common risk factors and shared pathophysiological mechanisms. The translation into the clinical practice of the significant advances in the comprehension of the underlying AF pathophysiology has been poor, as there is a lack of specific targeted treatments. Despite the numerous clinical trials that had addressed different aspects of treatment of patients with isolated HF or AF, few have focused on the management of patients with the combination of both diseases. Nevertheless, when managing a patient with HF and AF, the clinician should be aware of the prognostic significance and some therapeutic implications of this increasingly common disease combination.
